# Risk factors for recurrent attacks of wheeze in preschool children: a population-based cohort study in England

**DOI:** 10.1136/archdischild-2024-328375

**Published:** 2025-06-20

**Authors:** David Lo, Claire Lawson, Jonathan Broomfield, Clare Gillies, Sharmin Shabnam, Erol A Gaillard, Hilary Pinnock, Jennifer Quint

**Affiliations:** 1 Respiratory Sciences, University of Leicester, Leicester, UK; 2 Paediatric Respiratory Medicine, University Hospitals of Leicester NHS Trust, Leicester, UK; 3 Cardiovascular Sciences, University of Leicester, Leicester, UK; 4 Population Health Sciences, University of Leicester, Leicester, UK; 5 The University of Edinburgh Usher Institute of Population Health Sciences and Informatics, Edinburgh, UK; 6 School of Public Health, Imperial College London, London, UK

**Keywords:** Child Health, Epidemiology, Paediatrics, Respiratory Medicine

## Abstract

**Objective:**

To determine factors associated with recurrent attacks of acute wheeze in preschool children.

**Design:**

Retrospective cohort study.

**Setting:**

English primary electronic health data from the Clinical Practice Research Datalink linked with hospital data from Hospital Episode Statistics.

**Participants:**

42 820 children aged 5 years or under with at least one acute wheeze presentation between 1 January 2013 and 31 December 2014.

**Exposures:**

Demographic and clinical variables including age, sex, ethnicity, deprivation quintile, clinical comorbidities and previous asthma medication prescriptions and acute attacks were included in multivariable analyses.

**Main outcome measures:**

Further healthcare presentation with an acute wheeze/asthma attack within 12 months.

**Results:**

Almost 40% (16 962/42 820) of children had a further attack within 12 months. The strongest predictors were hospitalisation with the index episode (RR 1.42; 95% CI 1.39 to 1.45) and an attack in the previous year (1.27; 1.22 to 1.32). Male sex (RR 1.06; 95% CI 1.03 to 1.08), South Asian ethnicity (1.08; 1.04 to 1.12), atopy (1.21; 1.18 to 1.24), prematurity (1.09; 1.04 to 1.14), increasing reliever prescriptions (1.04; 1.03 to 1.04), number of previous attacks (1.03; 1.02 to 1.04) and previous hospitalisation with wheeze (1.09; 1.05 to 1.14) were also associated with further attacks.

Older age at presentation (RR 0.92; 0.91 to 0.93) and number of prescriptions for inhaled corticosteroids (0.96; 0.95 to 0.97) in the previous year were associated with lower risk for further attacks.

**Conclusions:**

Our findings can be used to aid clinical risk prediction for further attacks of wheeze in preschool children.

**WHAT IS ALREADY KNOWN ON THIS TOPIC**

- Acute presentations with wheeze are extremely common in preschool children.
- There is limited evidence to inform the risk of further attacks of wheeze in preschool children.
- This is important to inform decisions to initiate regular preventer asthma treatments in this age group.

**WHAT THIS STUDY ADDS**

- This is the first study to report risk factors associated with further attacks of wheeze in preschool children following an index presentation with wheeze to any care setting.

**HOW THIS STUDY MIGHT AFFECT RESEARCH, PRACTICE OR POLICY**

- Findings from this study can be used to inform clinical decision making and develop a risk stratification tool to personalise initiation and escalation of regular preventative wheeze/asthma treatment in preschool children.

## Background

 Wheezing illnesses are common in preschool children, with up to half experiencing wheeze by age 6 years.^1 2^ Most episodes are mild and secondary to viral respiratory infections, but some children experience multiple attacks or symptoms between acute infections resulting in frequent healthcare attendances and poor quality of life.^3 4^ These children are more likely to have asthma and may benefit from regular treatment with inhaled corticosteroids (ICSs). Unlike in older children and adults, objective testing to confirm asthma is not practical in preschool children, leading to hesitance with diagnosis and initiating treatment.^5^

Current guidelines recommend a trial of asthma preventer treatment in preschool children with severe or frequent wheezing, without confirmatory objective testing,^6 7^ with the caveat that a thorough clinical history should be undertaken to exclude alternative causes.

Despite the incidence of unscheduled healthcare attendances for acute wheeze in preschoolers increasing, there has been no evidence of a corresponding increase in ICS prescriptions from routine health data, but instead an overall increase in reliance on short-acting beta-2 agonists (SABAs) and short courses of oral corticosteroids.^8^ This reflects reports of asthma treatment being overly focused on managing acute presentations, with less emphasis on long-term preventative management.^9 10^

The assessment of future risk of acute attacks is an essential component of asthma care in any age group.^6 7^ In older children, a history of previous attacks, raised fraction of exhaled nitric oxide (FeNO) and increase in SABA use relative to ICS have been associated with increased risk for further attacks.^11 12^ There is limited evidence, however, to inform risk prediction in preschool children. This has been identified as a research need.^7^

We aimed to describe clinically relevant and patient demographic factors associated with further attacks of wheeze in preschool children who have been seen acutely with wheeze in either primary or secondary care.

## Methods

### Data source

In this cohort study, we used de-identified routine healthcare data from the Clinical Practice Research Datalink Aurum (CPRD-A) national primary care database, containing patient information from consenting UK general practices (GPs). The data set is broadly representative of the UK population in terms of age, sex and ethnicity;^13^ and contains coded data on patient demographics, diagnoses, prescriptions, tests and referrals. Data from the May 2022 build^14^ were linked via patient residential postcode to small-area level data^15^ for socioeconomic deprivation measures and rural–urban classification data.^16^ Linkage to secondary care data was available from NHS England’s Hospital Episode Statistics (HES) Admitted Patient Care,^17^ Outpatient^18^ and Accident and Emergency (A&E)^19^ databases.

### Population

The study population was children 5 years or under with at least one acute wheeze presentation to primary or secondary care between 1 January 2013 and 31 December 2014; registered with GPs in England contributing data to CPRD-A and eligible for HES linkage. These dates were chosen pragmatically based on the availability of data, which were requested as part of an earlier analysis to explore health inequalities in preschool wheeze as part of the same research programme.^20^

We excluded children with primary ciliary dyskinesia, cystic fibrosis, bronchopulmonary dysplasia, bronchiolitis obliterans, interstitial lung disease or bronchiectasis.

### Outcomes

The index date was defined as the date of the first episode of acute wheeze between 1 January 2013 and 31 December 2014. The main outcome of interest was a subsequent episode of acute wheeze, presenting to any setting within 12 months of the index date. The secondary outcome was a subsequent episode of acute wheeze requiring hospitalisation or critical care within 12 months.

Acute attendances were defined using clinical diagnostic ‘medcodeids’ (derived from Read and SNOMED (Systematized Nomenclature of Medicine Clinical Terms) codes) for primary care, and International Classification of Diseases, 10th Revision/A&E wheeze/asthma diagnostic or treatment codes for secondary care. For primary care data, we developed a broad list of >200 diagnostic and treatment codes to define our outcome, to reflect the large number of Read and SNOMED codes for wheeze and asthma available, and the variable use of these codes between practitioners. Code lists used are available via https://github.com/dkhl1/ip2am, and details on how these were created here: https://github.com/NHLI-Respiratory-Epi/SNOMED-CT-codelists.

### Variables

The following demographic data were extracted from CPRD and HES data sets: age at index date (whole years), sex, socioeconomic status, ethnicity and urban/rural classification of home postcode.

Socioeconomic status was based on the 2019 English Index of Multiple Deprivation (IMD) linked to the patient’s postcode, grouped into quintiles.^15^ Ethnicity was defined, from HES data sets, as the most commonly recorded ethnicity value across all healthcare episodes and grouped into five categories.

The rural–urban data set, produced by the UK’s Office for National Statistics using census data, was used to categorise patient residences as either primarily ‘rural’ or ‘urban’ at the patient postcode level. Areas are classified as rural if they fall outside of settlements with more than a 10 000-resident population.^21^

Clinical diagnostic codes were used to define the presence of clinical comorbidities including atopy (defined as the presence of eczema, hay fever, food allergy and/or rhinitis), gastro-oesophageal reflux, prematurity and history of bronchiolitis.

Data on previous healthcare utilisation and wheeze treatment prior to the index date were extracted: number of wheeze attacks ever, any attacks 12 months preindex date, previous hospitalisations with wheeze, setting of index episode (primary care, A&E, hospitalisation/critical care admission), number of reliever (SABA or ipratropium bromide) prescriptions 12 months preindex date and number of ICS prescriptions 12 months preindex date. Medication codes were used to determine reliever and ICS prescriptions from CPRD records.

### Statistical analyses

Frequency distributions of cohort characteristics were presented for sex, urban/rural classification, IMD quintile and ethnicity. Age at index episode, number of previous acute wheeze episodes, healthcare attendances within previous year for acute wheeze, previous hospital admissions for wheeze and number of reliever and ICS prescriptions in the previous year were summarised as: mean (SD) or median (25th, 75th percentiles) for continuous variables, and as counts and percentages for categorical and binary variables.

Cross tabulation was performed to present the number (proportion) of children with further attacks by each variable.

Separate Poisson generalised linear models with log link and robust standard errors (complete case) were fitted to explore associations between participant characteristics and the outcomes of interest. Potential risk factors were determined based on clinical relevance and existing literature, and only suitable predictors were considered for the analysis.

Adjusted relative risks (RRs) with associated 95% CIs are reported. The variance inflation factor (VIF) was used to check for collinearity between variables (Stata ‘collin’ command), with a value >10 suggesting evidence of collinearity.^22^

Significant associations should be interpreted in the context that numerous variables were assessed, increasing the probability of a type 1 error.

All analyses were performed using Stata statistical software, V.18.0.

### Patient and public involvement

Two patient and public involvement contributors with experience of caring for children with recurrent preschool wheeze were members of the project steering committee. They commented on the study protocol and have been consulted on the contextual interpretation of the study findings and dissemination strategies.

## Results

### Characteristics of children at index episode

The cohort included 42 820 preschool children and their characteristics are shown in table 1. Children with any missing data were low (2.6%), and the characteristics of children with complete data were similar to the whole cohort (online supplemental table S1).

**Table 1:** Baseline characteristics of cohort

Variable group	Variable categories	Total (n=42 820)
Age at index episode	Median (25th, 75th percentile) age in years	1 (1, 3)
Sex	Female	15 791 (36.9%)
	Male	27 029 (63.1%)
Urban or rural classification of household	Urban	37 567 (87.7%)
	Rural	5215 (12.2%)
	Missing/unknown	38 (0.1%)
2019 English IMD Quintiles (1=least deprived)	1	7568 (17.6%)
	2	7321 (17.1%)
	3	7664 (17.9%)
	4	9107 (21.3%)
	5	11 122 (26.0%)
	Missing/unknown	38 (0.1%)
Recorded ethnicity	White	31 433 (73.4%)
	South Asian	4838 (11.3%)
	Black	2446 (5.7%)
	Other	1102 (2.6%)
	Mixed	1922 (4.5%)
	Missing/unknown	1079 (2.5%)
Comorbidities	History of atopy (including food allergy, eczema, hay fever, rhinitis)	21 445 (50.1%)
	History of prematurity (born at <37 weeks gestation)	2499/42 820 (5.8%)
Medication prescriptions in 12 months prior to index date	Median (25th, 75th percentile) (range) number of relievers	0 (0, 1) (0–33)
	Median (25th, 75th percentile) (range) number of ICS	0 (0, 0) (0–18)
Healthcare setting of index episode	Primary care	25 292 (59.1%)
	Emergency department	4675 (10.9%)
	Hospital admission	12 851 (30.0%)
	Critical care admission	2 (0.0%)
Previous attacks of wheeze	Median number of attacks ever prior to index episode (25th, 75th percentile) (range)	0 (0, 0) (0–58)
	History of at least one hospitalisation ever prior to index episode	4793/42 820 (11.2%)
	History of at least one attack within 12 months prior to index episode	4358/42 820 (10.2%)

ICS, inhaled corticosteroid; IMD, index of multiple deprivation.

The majority of children were male (27 029; 63.1%), lived in an urban area (37 567; 87.7%) and were of white ethnicity (31 433; 73.4%). Children from the most deprived two quintiles made up almost half of the cohort (20 229; 47.3%). Half of the children had evidence of atopy (21 445; 50.1%), and 2499 (5.8%) were born preterm (<37 weeks gestation).

Of the index episodes, the majority were managed in primary care (25 292; 59.1%), and around a third were hospitalised (12 851; 30.0%). One-in-10 children (4358; 10.2%) had at least one attack 12 months preindex, and 9041 (21.1%) had at least one attack ever preindex episode (online supplemental table S2).

**Figure 1:**
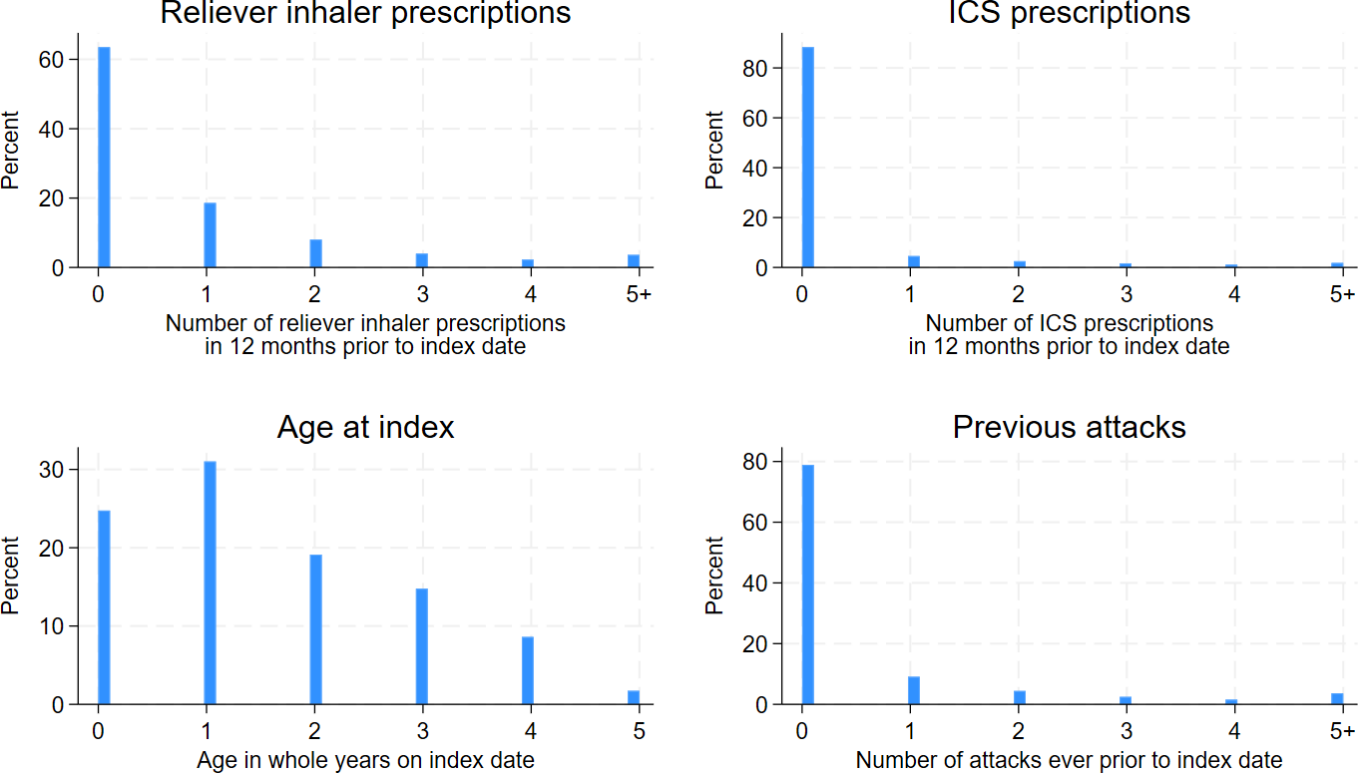
Histogram showing frequency distribution of continuous variables. ICS, inhaled corticosteroids.

One-third of the cohort (15 622; 36.5%) had been prescribed at least one reliever inhaler in the year preindex attack, with 1548 (3.6%) prescribed five or more (online supplemental table S3). 4995 (11.7%) children were prescribed at least one ICS inhaler in the year preindex attack (online supplemental table S4). The distribution of children’s ages, number of previous attacks and number of prescriptions for reliever or ICS medications are shown in figure 1.

### Risk factors for further presentation with acute wheeze

Almost 40% (16 962/42 820) of our cohort had a further attack of wheeze within a year of the index episode. This increased to 61% (944/1553) in children with five or more attacks ever preindex episode, compared with 38% (12 683/33 779) in those presenting with their first attack; and to 58% (2516/4358) in children with at least one attack in the previous year, compared with 38% (14 446/38 462) in those with no attacks in the previous year (table 2).

**Table 2:** Cross-tabulation of primary outcome (further attacks or not 12 months postindex) by each available variable

Variable group	Variable categories	Further attacks 12 months postindex		Total
		Yes n=16 962 (39.6%)	No n=25 858 (60.4%)	
Age at index episode (years)	0	4258 (40.2%)	6339 (59.8%)	10 597
	1	5335 (40.2%)	7947 (59.8%)	13 282
	2	3429 (41.9%)	4761 (58.1%)	8190
	3	2443 (38.6%)	3883 (61.4%)	6326
	4	1306 (35.4%)	2380 (64.6%)	3686
	5	191 (25.9%)	548 (74.1%)	739
Sex	Female	5889 (37.3%)	9902 (62.7%)	15 791
	Male	11 073 (41.0%)	15 956 (59.0%)	27 029
Urban or rural classification of household	Urban	14 853 (39.5%)	22 714 (60.5%)	37 567
	Rural	2098 (40.2%)	3117 (59.8%)	5215
	Missing/unknown	11 (29.0%)	27 (71.0%)	38
2019 English IMD Quintiles (1=least deprived)	1	2958 (39.1%)	4610 (60.9%)	7568
	2	2890 (39.5%)	4431 (60.5%)	7321
	3	3021 (39.4%)	4643 (60.6%)	7664
	4	3654 (40.1%)	5453 (59.9%)	9107
	5	4428 (39.8%)	6694 (60.2%)	11 122
	Missing/unknown	11 (29.0%)	27 (71.0%)	38
Recorded ethnicity	White	12 367 (39.3%)	19 066 (60.7%)	31 433
	South Asian	2082 (43.0%)	2756 (57.0%)	4838
	Black	984 (40.2%)	1462 (59.8%)	2446
	Other	443 (40.2%)	659 (59.8%)	1102
	Mixed	807 (58.0%)	1115 (42.0%)	1922
	Missing or unknown	279 (25.9%)	800 (74.1%)	1079
History of atopy (including food allergy, eczema, hay fever, rhinitis)	Yes	9330 (43.5%)	12 115 (56.5%)	21 445
	No	7632 (35.7%)	13 743 (64.3%)	21 375
History of prematurity (born at <37 weeks gestation)	Yes	1101 (44.1%)	1398 (55.9%)	2499
	No	15 861 (39.3%)	24 460 (60.7%)	40 321
Number of reliever prescriptions 12 months preindex	0	9911 (36.4%)	17 287 (63.6%)	27 198
	1–2	4931 (43.2%)	6472 (56.8%)	11 403
	3–4	1274 (47.7%)	1397 (52.3%)	2671
	5+	846 (54.7%)	702 (45.4%)	1548
Number of ICS prescriptions 12 months preindex	0	14 679 (38.8%)	23 146 (61.2%)	37 825
	1–2	1363 (44.9%)	1671 (55.1%)	3034
	3–4	555 (46.7%)	634 (53.3%)	1189
	5+	365 (47.3%)	407 (52.7%)	772
Healthcare setting of index episode	Primary care	8782 (34.7%)	16 510 (65.3%)	25 292
	Emergency department	1836 (39.3%)	2839 (60.7%)	4675
	Hospital admission	6343 (49.4%)	6508 (50.6%)	12 851
	Critical care admission	1 (50.0%)	1 (50.0%)	2
Number of attacks ever preindex	0	12 683 (37.6%)	21 096 (62.4%)	33 779
	1–2	2444 (42.3%)	3332 (57.7%)	5776
	3–4	891 (52.0%)	821 (48.0%)	1712
	5+	944 (60.8%)	609 (39.2%)	1553
History of at least one hospitalisation ever prior to index episode	Yes	2532 (52.8%)	2261 (47.2%)	4793
	No	14 430 (38.0%)	23 597 (62.0%)	38 027
History of at least one attack within 12 months prior to index episode	Yes	2516 (57.7%)	1842 (42.3%)	4358
	No	14 446 (37.6%)	24 016 (62.4%)	38 462

ICS, inhaled corticosteroid; IMD, index of multiple deprivation.

**Figure 2:**
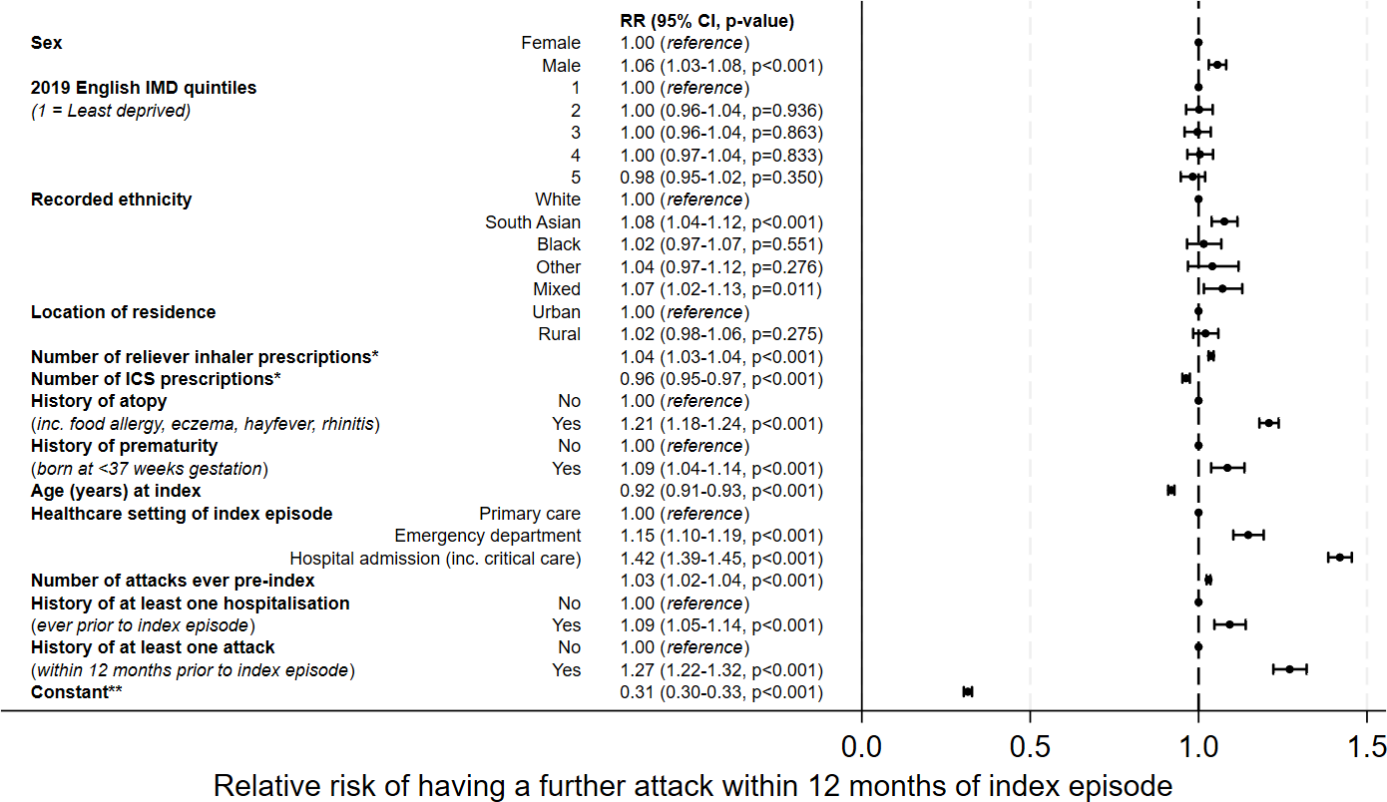
Forest plot of outputs from multivariable regression analysis for primary outcome, adjusted for all covariates presented. The relative risk (RR) is presented adjacent to each variable, followed by the 95% CI and p value. *Number of prescriptions in 12 months prior to index episode. **Constant estimate denotes the baseline risk of an event for an individual with all variables set to the reference level. ICS, inhaled corticosteroid; IMD, index of multiple deprivation.

Figure 2 shows the estimates of association between each variable and the RR of a subsequent wheeze attack within 12 months of the index episode. The strongest predictor for further attacks was hospitalisation at index episode (RR 1.42; 95% CI 1.39 to 1.45) and having at least one attack in the previous year (1.27; 1.22 to 1.32).

Other variables associated with further attacks were: history of atopy (RR 1.21; 95% CI 1.18 to 1.24), index episode treated in emergency department (ED) (1.15; 1.10 to 1.19), prematurity (1.09; 1.04 to 1.14), South Asian ethnicity (1.08; 1.04 to 1.12), previous hospitalisation with wheeze (1.09; 1.05 to 1.14), male sex (1.06; 1.03 to 1.08), increasing number of reliever prescriptions (1.04; 1.03 to 1.04) and increasing number of previous attacks (1.03; 1.02 to 1.04).

Conversely, older age at presentation and prescriptions for ICS were associated with lower risk for further attacks. For each year increase in age at index presentation, there was an 8% lower risk of a further attack (RR 0.92; 95% CI 0.91 to 0.93). Each additional ICS prescription in the year prior to the index attack reduced the risk of a subsequent attack by 4% (0.96; 0.95 to 0.97).

Neither deprivation quintile nor urbanicity of home postcode was associated with the risk of further attacks.

### Risk factors for further presentation with acute wheeze requiring hospitalisation

**Figure 3:**
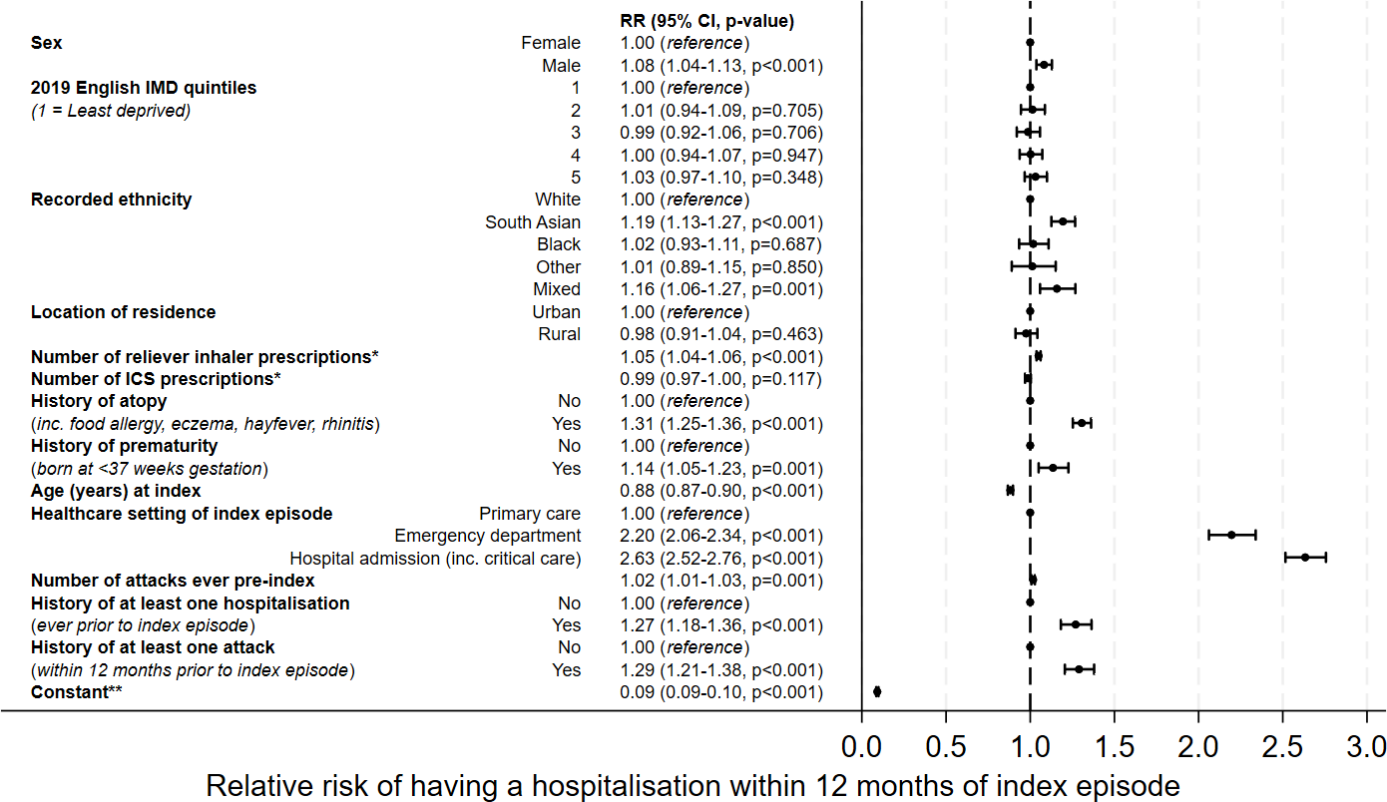
Forest plot of outputs from multivariable regression analysis for primary outcome, adjusted for all covariates presented. The relative risk (RR) is presented adjacent to each variable, followed by the 95% CI and p value. *Number of prescriptions in 12 months prior to index episode. **Constant estimate denotes the baseline risk of an event for an individual with all variables set to the reference level. ICS, inhaled corticosteroid; IMD, index of multiple deprivation.

Figure 3 shows the estimates of association between each variable and the RR of a severe attack requiring hospitalisation within 12 months of the index episode. Male sex, South Asian ethnicity, atopy, prematurity, increasing number of reliever prescriptions, increasing number of previous attacks, previous hospitalisation with wheeze, an attack within the previous year and care for the index episode either in ED or hospital were all associated with increased risk of further attack. The index episode presenting in ED (RR 2.20; 95% CI 2.06 to 2.34) or requiring hospitalisation (2.63; 2.52 to 2.76) was associated with a greater than twofold increase in the risk of hospitalisation with wheeze within a year.

Older age at index presentation was also associated with lower risk for further severe attacks, but increased prescriptions of ICS were not associated with a reduction in severe attacks. Neither deprivation quintile nor urbanicity of home postcode was associated with further severe attacks.

In our analysis, the mean VIF was 1.35 (maximum VIF 1.95), indicating no evidence of significant multicollinearity between variables, including between number of wheeze attacks ever and any attacks within the year preindex date. There was no notable evidence of non-linearity observed for the continuous variables included within the models. A graphical inspection of deviance and Pearson residual distributions against fitted values confirmed suitable model fit for both outcomes.

## Discussion

In this analysis of over 40 000 preschool children presenting to any healthcare setting with attacks of wheeze in England, almost 40% presented with a further attack within 12 months. This increased to almost 50% in children presenting to hospital, and to almost 60% in children with at least one further attack in the previous year. We report multiple factors associated with increased risk for a further attack, including severity of index presentation, history of previous attacks, atopy, premature birth, younger age at presentation, higher reliever inhaler usage and fewer inhaled steroid prescriptions in the previous year.

Our findings are comparable with previous studies in older children and adults.^11 12 23^ A 2018 systematic review of risk factors for asthma attacks in children aged 5–12 years reported the highest risk of further attacks to be in children with a history of previous asthma attacks (ORs between 2.0 and 4.1). In our younger cohort, a previous attack is also associated with further attacks, but the association is less strong compared with older children. This may be because asthma is more likely to be the cause for acute wheeze in older children, while in children under 5, wheeze is more commonly a presenting feature of self-limiting viral chest infections; so-called ‘viral-induced wheeze’.

In preschool children, we observed the strongest predictor for a further attack was severity of the index presentation. Children requiring hospitalisation for acute wheeze were almost 1.5 times as likely to have a further attack compared with children requiring treatment in primary care only. The severity of the index presentation was also associated with the risk of subsequent hospitalisation with wheeze within 12 months. Preschool children cared for in hospital or the ED have over double the risk of hospitalisation within 12 months compared with children who only required treatment in primary care.

Existing evidence to inform risk prediction in preschool children is limited.^7^ Similar to our study, a previous large UK cohort study using routinely collected healthcare data (n=17 320 preschool children)^24^ reported the following risk factors for further attacks in preschool children: younger age, atopic disease and male sex. Interestingly, and in contradiction to our findings, their study reported low socioeconomic status and treatment with regular preventer medication to be associated with higher risk of asthma attacks; each increasing British Thoracic Society (BTS) 2016 asthma guideline treatment step was associated with incremental increases in the risk of future attacks. However, their study only included children with a coded diagnosis of asthma, thus likely only including children with better access to GPs (where most diagnoses are made) and excluding children with poor access to primary healthcare services, including those from more deprived backgrounds.^20^ Asthma treatment step was defined as the highest BTS step (highest asthma medication dosage) based on all medications prescribed in the year prior to study entry. Information on the number of preventer inhalers prescribed (as a surrogate indicator for adherence), reliever prescriptions (symptom control) or history of previous attacks was not available in their analysis. After adjusting for these variables within our analysis, increasing inhaled steroid prescriptions was found to be associated with reduced risk of further attacks, which would intuitively make sense as previous studies have demonstrated a positive response to inhaled steroids in at least a subgroup of preschool children with recurrent wheeze.^25 26^

We chose not to limit our cohort to include only preschool children with a coded ‘asthma diagnosis’ within their electronic health records. Symptom overlap with other self-limiting respiratory conditions and the lack of practical objective tests means diagnosing asthma in preschool children is not straightforward. For our analysis, we wanted findings to be generalisable to any preschool child presenting acutely to healthcare with wheeze, and not limit risk prediction to only those with a pre-existing physician diagnosis.

Other key strengths of our study are its large, representative sample size and use of standardised clinical codes for case finding and outcome definitions. Linkage to emergency department and hospital discharge data further allowed us to capture episodes regardless of healthcare setting.

### Limitations

Healthcare data is not primarily collected or curated for research purposes. Clinical coding is therefore not standardised between health professionals, which may result in data inaccuracies. To avoid missing potential acute wheeze episodes, we incorporated broad clinical codes for acute wheeze and asthma to account for clinician differences in diagnostic recording.

The number of prescriptions for both inhaled steroids and asthma relievers was likely underestimated as we were only able to include medication prescriptions issued from primary care, and not those from hospital or A&E.

We decided to include the number of ICS prescriptions in our analysis as a surrogate indicator for adherence. However, electronic prescriptions do not necessarily represent what was dispensed by pharmacies or what was used by patients, potentially over-estimating ICS and/or reliever usage.

Predictors included in our analysis were limited to those which were consistently recorded and available in the data sets used. Other previously reported biomarkers for future attacks, such as FeNO or low lung function, were not available to us. Similarly, predictors for response to regular inhaled steroids, such as blood eosinophils, are not routinely performed within the primary care setting in preschool children, and not available from HES.

Finally, the data are from over 10 years ago, due to this being a secondary analysis of data requested for a previous analysis. We are confident, however, that our findings remain valid as the association between our outcomes of interest with the available demographic and predictor variables included is unlikely to have changed since 2013, particularly since there have been no radical changes to asthma guidelines pertaining to preschool children over this time period. Moreover, by using broad clinical codes for attacks (including those for both wheeze and asthma) and linking both primary and secondary care data sets, our findings will remain relevant even if there have been changes in how asthma attacks are coded or which healthcare settings children are taken to for attacks over the past decade.

## Conclusions

The aim of asthma treatment is not only to improve symptom control, but also to reduce the risk of further attacks. In this study, we report clinically relevant risk factors associated with further attacks in children presenting acutely with an asthma/wheeze attack. While no single factor can predict a further attack with complete certainty, children presenting to hospital and those who have had an attack within the previous year have a 40% and almost 30% higher risk respectively for a further attack in the following year, and warrant closer follow-up and monitoring, and consideration for regular asthma medications.

Building on our present analysis, we plan to develop a clinical prediction tool to improve further the accuracy of risk stratification in preschool children with wheeze.

## Supplementary material

10.1136/archdischild-2024-328375 online supplemental file 1

## Data Availability

Data may be obtained from a third party and are not publicly available.
