# Supplementary material for: Risk factors for recurrent attacks of wheeze in preschool children: a population-based cohort study in England
Source: Arch Dis Child. 2025 Jun 20;110(11):e328375. doi: 10.1136/archdischild-2024-328375 (PMC12573339; doi:10.1136/archdischild-2024-328375)
Supplement: online supplemental file 1 [file archdischild-110-11-s001.docx]

**SUPPLEMENTARY FILE**

Table S1. Baseline characteristics of cohort according to missing data status

| **Variable Group** | **Variable Categories** | **Whole cohort** | **Complete case** | **Incomplete case** |
| --- | --- | --- | --- | --- |
|  |  | N = 42,820 | N = 41,706 | N = 1,114 |
| **Age at Index Episode** | Median (25^th^, 75^th^ percentile) age in years | 1 (1, 3) | 1 (1, 3) | 1 (0, 3) |
| **Sex** | Female | 15,791 (36.9%) | 15,340 (36.8%) | 451 (40.5%) |
|  | Male | 27,029 (63.1%) | 26,366 (63.2%) | 663 (59.5%) |
| **Co-morbidities** | History of Atopy (inc. food allergy, eczema, hayfever, rhinitis) | 21,445 (50.1%) | 20,949 (50.2%) | 496 (44.5%) |
|  | History of Prematurity (born at <37 weeks gestation) | 2,499 (5.8%) | 2,455 (5.9%) | 44 (4.0%) |
| **Medication Prescriptions in 12 months Prior to Index Date** | Median (25^th^, 75^th^ percentile) number of Relievers | 0 (0, 1) | 0 (0, 1) | 0 (0, 1) |
|  | Median (25^th^, 75^th^ percentile) number of ICS | 0 (0, 0) | 0 (0, 0) | 0 (0, 0) |
| **Healthcare Setting of Index Episode** | Primary Care | 25,292 (59.1%) | 24,405 (58.5%) | 887 (79.6%) |
|  | Emergency Department | 4,675 (10.9%) | 4, 573 (11.0%) | 102 (9.2%) |
|  | Hospital Admission | 12,851 (30.0%) | 12,726 (30.5%) | 125 (11.2%) |
|  | Critical Care Admission | <5 (0.0%) | <5 (0.0%) | 0 (0.0%) |
| **Previous Attacks of Wheeze** | Median number of attacks ever prior to index episode (25^th^, 75^th^ percentile) | 0 (0, 0) | 0 (0, 0) | 0 (0, 0) |
|  | History of at least one hospitalisation ever prior to index episode | 4,793 (11.2%) | 4,762 (11.4%) | 31 (2.8%) |
|  | History of at least one attack within 12 months prior to index episode | 4,358 (10.2%) | 4,305 (10.3%) | 53 (4.8%) |

The table above presents the baseline characteristics of the cohort stratified across whether children had any missing data or not. This demonstrates the representativeness of the complete case cohort, with any differences in variable groups likely to be down to the small number of children with incomplete case data, and justifies the choice of a complete case analysis.

Table S2. Frequency distribution of number of attacks with wheeze ever prior to index episode

| **Number of Wheeze Attacks Ever Prior to Index Episode** | **Frequency** | **Percentage** | **Cumulative Percentage** |
| --- | --- | --- | --- |
| 0 | 33,779 | 78.9 | 78.9 |
| 1-2 | 5,776 | 13.5 | 92.4 |
| 3-4 | 1,712 | 4.0 | 96.4 |
| 5-6 | 730 | 1.7 | 98.1 |
| 7-8 | 376 | 0.9 | 99.0 |
| 9-10 | 176 | 0.4 | 99.4 |
| 11-12 | 95 | 0.2 | 99.6 |
| >12 | 176 | 0.4 | 100 |
| **Total** | 42,820 | 100 |  |

Table S3. Frequency distribution of asthma reliever prescriptions in the previous year

| **Number of Relievers Prescribed in Previous Year** | **Frequency** | **Percentage** | **Cumulative Percentage** |
| --- | --- | --- | --- |
| 0 | 27,198 | 63.5 | 63.5 |
| 1-2 | 11,403 | 26.6 | 90.1 |
| 3-4 | 2,671 | 6.2 | 96.4 |
| 5-6 | 847 | 2.0 | 98.4 |
| 7-8 | 352 | 0.8 | 99.2 |
| 9-10 | 162 | 0.4 | 99.6 |
| 11-12 | 91 | 0.2 | 99.8 |
| >12 | 96 | 0.2 | 100 |
| **Total** | 42,820 | 100 |  |

Table S4. Frequency distribution of inhaled corticosteroid (ICS) prescriptions in the previous year

| **Number of ICS Prescribed in Previous Year** | **Frequency** | **Percentage** | **Cumulative Percentage** |
| --- | --- | --- | --- |
| 0 | 37,825 | 88.3 | 88.3 |
| 1-2 | 3,034 | 7.1 | 95.4 |
| 3-4 | 1,189 | 2.8 | 98.2 |
| 5-6 | 425 | 1.0 | 99.2 |
| 7-8 | 183 | 0.4 | 99.6 |
| 9-10 | 86 | 0.2 | 99.8 |
| 11-12 | 56 | 0.1 | 99.9 |
| >12 | 22 | 0.1 | 100 |
| **Total** | 42,820 | 100 |  |
